# Social determinants of multimorbidity in Jamaica: application of latent class analysis in a cross-sectional study

**DOI:** 10.1186/s12889-021-11225-6

**Published:** 2021-06-23

**Authors:** Leslie S. Craig, Colette A. Cunningham-Myrie, David R. Hotchkiss, Julie H. Hernandez, Jeanette Gustat, Katherine P. Theall

**Affiliations:** 1 Department of Medicine, School of Medicine, Tulane University, New Orleans, LA USA; 2 Department of Community Health & Psychiatry, University of the West Indies, Mona, Jamaica; 3 Department of Global Community Health and Behavioral Sciences, School of Public Health and Tropical Medicine, Tulane University, New Orleans, LA USA; 4 Department of Health Policy and Management, School of Public Health and Tropical Medicine, Tulane University, New Orleans, LA USA; 5 Department of Epidemiology, School of Public Health and Tropical Medicine, Tulane University, New Orleans, LA USA

**Keywords:** Non-communicable diseases, Multimorbidity, Social determinants, Jamaica, Latent class analysis

## Abstract

**Background:**

Non-communicable disease (NCD) multimorbidity is associated with impaired functioning, lower quality of life and higher mortality. Susceptibility to accumulation of multiple NCDs is rooted in social, economic and cultural contexts, with important differences in the burden, patterns, and determinants of multimorbidity across settings. Despite high prevalence of individual NCDs within the Caribbean region, exploration of the social epidemiology of multimorbidity remains sparse. This study aimed to examine the social determinants of NCD multimorbidity in Jamaica, to better inform prevention and intervention strategies.

**Methods:**

Latent class analysis (LCA) was used to examine social determinants of identified multimorbidity patterns in a sample of 2551 respondents aged 15–74 years, from the nationally representative Jamaica Health and Lifestyle Survey 2007/2008. Multimorbidity measurement was based on self-reported presence/absence of 11 chronic conditions. Selection of social determinants of health (SDH) was informed by the World Health Organization’s Commission on SDH framework. Multinomial logistic regression models were used to estimate the association between individual-level SDH and class membership.

**Results:**

Approximately one-quarter of the sample (24.05%) were multimorbid. LCA revealed four distinct profiles: a *Relatively Healthy* class (52.70%), with a single or no morbidity; and three additional classes, characterized by varying degrees and patterns of multimorbidity, labelled *Metabolic* (30.88%), *Vascular-Inflammatory* (12.21%), and *Respiratory* (4.20%). Upon controlling for all SDH (Model 3), advancing age and recent healthcare visits remained significant predictors of all three multimorbidity patterns (*p* < 0.001). Private insurance coverage (relative risk ratio, RRR = 0.63; *p* < 0.01) and higher educational attainment (RRR = 0.73; *p* < 0.05) were associated with lower relative risk of belonging to the *Metabolic* class while being female was a significant independent predictor of *Vascular-Inflammatory* class membership (RRR = 2.54; *p* < 0.001). Material circumstances, namely housing conditions and features of the physical and neighbourhood environment, were not significant predictors of any multimorbidity class.

**Conclusion:**

This study provides a nuanced understanding of the social patterning of multimorbidity in Jamaica, identifying biological, health system, and structural determinants as key factors associated with specific multimorbidity profiles. Future research using longitudinal designs would aid understanding of disease trajectories and clarify the role of SDH in mitigating risk of accumulation of diseases.

## Background

Social conditions influence behavioural risk factors for non-communicable diseases (NCDs) and consequent intermediate changes in body weight and body composition (i.e., metabolic risk factors), in addition to health-seeking behaviours and opportunities to intervene in the onset, expression and outcome of disease [1–3]. Individually, NCDs are responsible for substantial death and disability [2]; importantly, when two or more NCDs occur together (i.e., multimorbidity), health outcomes are further modified, often through the decreased quality of life that results from increased burden of diseases, increased health care consumption and reduced coping strategies [4, 5].

Multimorbidity has typically been studied in high-income countries across Europe, North America and Australia, where its prevalence and socio-economic determinants have been well established [6–8]. In a recent systematic review, significant positive associations with multimorbidity prevalence were consistently reported, across 44 studies from high-income settings, with advancing age (odds ratios [ORs] ranging from 1.26 to 227.46) and lower socio-economic status (with ORs ranging from 1.20 to 1.91), while some sites additionally reported associations between being female and the presence of mental health disorders [6]. Notably, while data from high-income settings has suggested that multimorbidity is associated with socio-economic deprivation [6], evidence from low- and middle-income country settings suggests an inverse relationship, with greater likelihood of reporting multimorbidity among individuals with higher per capita household income in China [9] and South Africa [10]. Together, these data suggest that while vulnerability to multimorbidity may be heightened by the common aetiology and shared pathogenesis of many NCDs, susceptibility is further conditioned by the environment in which people live and work, their adaptive capacities and their behavioural risk factors [3, 4].

**Fig. 1:**
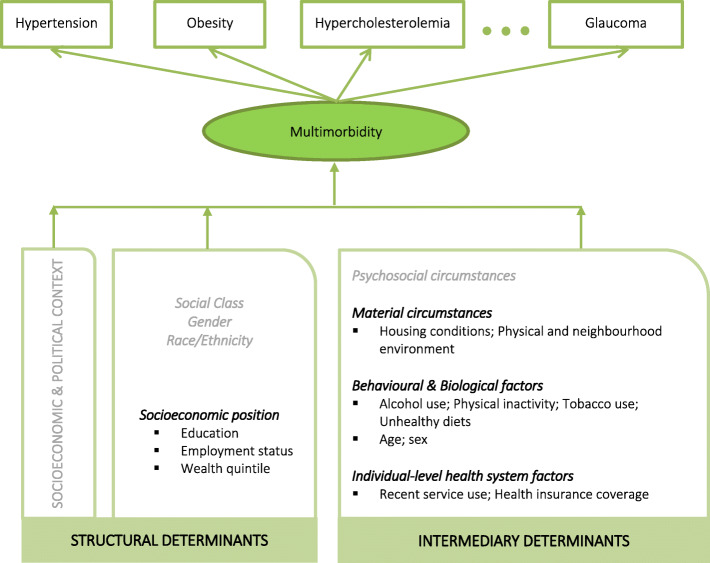
Conceptual framework for analysis of multimorbidity and its relationship with social determinants of health (SDH)

Relationships between behavioural risk factors (e.g., tobacco use, physical activity, unhealthy diets) and prevalence of individual chronic conditions (e.g., diabetes, hypertension) have been reported elsewhere using nationally representative data from the Jamaica Health and Lifestyle Survey (JHLS) 2000/2001 [11, 12] and the JHLS 2007/2008 [13–15]. However, no investigation of the social determinants of specific patterns or combinations of diseases has been undertaken to date in Jamaica. Recent work using latent class analysis (LCA) points to a high prevalence of multimorbidity in the Jamaican population, that is disproportionately borne by women, and comprises three distinct multimorbidity patterns (labelled *Metabolic*, *Vascular-Inflammatory*, and *Respiratory*), in addition to a *Relatively Healthy* class characterized by little to no morbidity [16]. LCA provides a novel approach to reducing data complexity and has been widely used in prevention research to identify underlying subgroups of individuals characterized by multiple dimensions (e.g., with similar disease probability profiles) [17]. The present research builds on that earlier work, examining the association between social determinants of health (SDH) and multimorbidity class membership, via analyses guided by a latent class model and the World Health Organization (WHO) Commission on Social Determinants of Health (CSDH) framework [3] (Fig. 1).

According to the CSDH model, structural determinants (i.e., social, economic and political context) and mechanisms that define individual socio-economic position (e.g., income, education, occupation, social class, gender, race/ethnicity) together with intermediary determinants (i.e., psychosocial circumstances/stressors, material circumstances, behavioural and biological factors, health system factors reflecting access to care) engender differential exposure and vulnerability to the occurrence and intensity of ill-health and its consequences [3]. Sex is presented in this conceptual model as a biological factor and intermediary determinant. Gender, on the other hand, is noted together with other key structural determinants (e.g., social class, race/ethnicity) to reflect social constructs that can often be linked to discrimination and reinforcement of social hierarchies [3]. As depicted in Fig. 1, multimorbidity will first be measured using observed indicator (i.e., NCD) variables (e.g., hypertension) and then, via examination of structural and intermediary determinants, this study will describe social factors associated with specific patterns of NCDs in Jamaica, as this knowledge may better inform prevention and intervention strategies.

## Methods

### Data

The JHLS 2007/2008 (i.e., JHLS-II) recruited a sample of 2848 Jamaicans, between 15 and 74 years, over a four-month period spanning from November 2007 and March 2008 [13, 14]. The study employed a multi-stage cluster sampling design, with participant recruitment based on a random selection of clusters (enumeration districts) proportionate to the size of the population within the 14 parishes of Jamaica [13]. Within each cluster, a random starting point was chosen and every 10th household systematically identified, with a single individual from each household being invited to participate [13]. Self-reported data on demographic characteristics, medical history and health behaviours were obtained via an interviewer-administered questionnaire and, guided by standardized protocols, objective physical (i.e., height, body weight, hip circumference, waist circumference) and biological (i.e., blood pressure, blood glucose, total cholesterol) assessments were made [13, 14]. Additional details on the study protocol, design, sampling and data collection strategies are provided in the technical report [13].

### Measures

Variables used in this study included indicators of multimorbidity class membership (i.e., individual NCDs) in addition to structural and intermediary determinants of health that were examined as predictors of class membership.

#### Multimorbidity

Indicators of multimorbidity class membership are all fully described elsewhere [16]. Briefly, selection of indicators for multimorbidity measurement was guided by criteria outlined in a 2011 systematic review [18], being limited to NCDs with the highest prevalence in this population (i.e., at least 1% in each sex) and grouping related conditions such as cardiovascular disease (i.e., heart disease, myocardial infarction, and circulation problems) and mental health disorders (i.e., depression, anxiety, psychosis, and other mental health problems) to enhance data quality. Self-reported bronchitis/pneumonia served as a proxy measure of chronic obstructive pulmonary disease (COPD). Disease presence or absence was based on self-report, for all but four conditions (i.e., obesity, hypertension, diabetes, hypercholesterolemia) where physical assessments were available and used to increase measurement validity and reliability. Obesity (i.e., BMI ≥ 30 kg/m^2^ based on height and weight measurements), diabetes (i.e., fasting plasma glucose value ≥7.0 mmol/L or self-reported use of medication for raised blood glucose), hypertension (i.e., systolic blood pressure ≥ 140 mmHg and/or diastolic blood pressure ≥ 90 mmHg or self-reported use of medication to lower blood pressure) and hypercholesterolemia (i.e., total cholesterol level 5.2 mmol/l or higher or self-reported use of medication to control blood cholesterol) definitions were based on WHO criteria [2]. The final list of conditions included hypertension, obesity, hypercholesterolemia, diabetes, asthma, arthritis, cardiovascular disease, mental health disorders, COPD, stroke, and glaucoma. Of note, obesity was included as a chronic condition given its recognition as a chronic disease requiring intervention [19], its inclusion in previous studies of multimorbidity indices and patterns [7, 18] and ability to corroborate findings in baseline LCA models with and without obesity, in our previous work [16].

#### Social determinants of health (SDH)

SDH indicators are described in Table 1 and the text below.

**Table 1:** Definitions of social determinants of health (SDH) variables

SDH domain	Variable definition
*Structural determinants of socio-economic position*	
Occupational status	Employed full-time (yes/no)
Educational attainment	Attained at least secondary level education or equivalent (yes/no)
Income level	Top 60% vs. bottom 40% wealth quintiles, based on principal components analysis of ownership of non-productive assets (e.g., phone, television) and living conditions (e.g., number of people per room)
*Intermediary determinants*	
**Material circumstances**	
Housing conditions	Summed score reflecting interviewer’s assessment of more favourable home environments (based on the physical condition, internal cleanliness and external appearance of the home, and physical condition of furnishings)
Place to obtain fruit and vegetables	Interviewer’s assessment of whether a place to obtain fresh fruits and vegetables was within walking distance of the participant’s home.
Recreational area in walking distance	Interviewer’s assessment of whether parks and recreation areas were within walking distance of the home
Perceived safety	Greater (i.e., very safe or safe) vs. lesser (i.e., usually safe, can be dangerous, very dangerous) perceived safety
Neighbourhood infrastructure	Summed score reflecting interviewer’s assessment of the presence or absence of specific features in the respondent’s community (e.g., paved roads, sidewalks, electricity supply to homes, telephone lines to homes, street lighting, clean streets, recreation areas/playing field/open spaces)
**Behavioural risk factors**	
Alcohol use	Currently use alcohol (yes/no)
Tobacco smoking	Past or present smoker (yes/no)
Physical activity	Low (i.e., no activity or some activity reported but not enough to meet moderate or high levels) vs. not low (i.e., ≥ 5 days of any combination of walking, moderate- or vigorous-intensity activities achieving a minimum of at least 600 MET-minutes/week) based on International Physical Activity Questionnaire (IPAQ)- Short Form guidelines
Excessive fast-food consumption	Self-reported eating at fast food places such as Burger King, KFC, Tastee, Juici Patties or Pizza Hut at least twice per week
Sugar-sweetened beverage (SSB) intake	Self-reported drinking of lemonade/soda/box drink at least daily
**Biological factors**	
Sex	Male vs. female
Age	Years of age (from 15 to 74 years)
**Health system factors**	
Insurance coverage	Ownership of private insurance
Recent health service use	Last blood pressure measurement was less than 6 months ago

#### Structural determinants of socio-economic position

In keeping with the CSDH framework, occupational status, educational attainment and income level were used to operationalize socio-economic position [3]. Binary indicators of occupational status and educational attainment were used to classify participants as being employed full-time (yes/no) and having attained at least secondary level education or equivalent (yes/no), respectively. Principal component analysis (PCA) was used to generate a wealth index, based on (yes/no) responses to several questions on household assets (i.e., ownership of gas/electric stove, refrigerator or freezer, microwave oven, telephone, radio, television set, cable, satellite dish, bicycle, motorbike, car, computer, washing machine, sewing machine, fan, air conditioner, compact disk (CD) player, stereo equipment, record player, and video cassette recorder) and living conditions (i.e., number of members per sleeping room). The wealth variable was categorized into quintiles (i.e., poorest to wealthiest) and dichotomized to reflect those in the top 60% and bottom 40% wealth quintiles. Gender and social class could not be operationalized in this study. Data on race/ethnicity were not included, though available, since the majority of the sample population (93.79%) identified as black.

#### Intermediary determinants

##### Material circumstances

- Housing conditions
  A housing conditions indicator was created by summing scores reflecting the interviewer’s assessment (rated from 1-excellent to 4-poor) of specific characteristics of the respondent’s home (i.e., the physical condition of the home; internal cleanliness of the home, physical condition of the furnishings, external appearance of the home). The Cronbach’s alpha internal consistency coefficient of this home quality summated scale was 0.96, exceeding the minimum acceptable value of 0.70 [20]. Items were reversed scored so that higher scores were indicative of more favourable home environments.
- Physical and neighbourhood environment
  Guided by Kerr and colleagues (2012) theoretical model of the role of neighbourhood environments in supporting positive health outcomes, four specific aspects of the built and social environment were examined to reflect: (1) walkability (i.e., ease of access to destinations); (2) access to parks and recreational amenities; (3) safety; and (4) aesthetics, including the availability of sidewalks and pleasant scenery [21]. The binary variable for walkability reflected the interviewer’s assessment of whether a place to obtain fresh fruits and vegetables was within walking distance of the participant’s home. A similar variable was created for access to parks and recreational amenities to reflect the interviewer’s assessment of whether recreation areas were within walking distance of the home. Perceived safety of the community was assessed using the question “How safe is it to walk in your community”, with responses on a 5-item scale ranging from very safe (1) to very dangerous (5). Responses were collapsed to create a dichotomous indicator of greater and lesser perceived safety. For aesthetics, an index was created to reflect the neighbourhood infrastructure. Specifically, interviewer responses regarding the presence or absence of specific features in the respondent’s community (e.g., paved roads, sidewalks) were summed. The Cronbach’s alpha internal consistency coefficient of the neighbourhood infrastructure scale was 0.71, with higher overall scores indicative of more favourable circumstances.

##### Behavioural risk factors

Dichotomous indicators of tobacco smoking, and alcohol use were created to reflect past or current tobacco use (yes/no) and current alcohol drinkers (yes/no), respectively. Physical activity levels were determined based on responses to the International Physical Activity Questionnaire (IPAQ)- Short Form [22]. In accordance with data cleaning guidelines for the IPAQ short form [22], metabolic equivalent (MET) levels were determined using scores on walking, moderate-intensity activity and vigorous-intensity activity, and used to create categories based on established cut-offs, reflecting high (i.e., ≥ 7 or more days of any combination of walking, moderate- or vigorous-intensity activities accumulating at least 3000 MET-minutes/week), moderate (i.e., ≥ 5 days of any combination of walking, moderate- or vigorous-intensity activities achieving a minimum of at least 600 MET-minutes/week) or low (i.e., no activity or some activity reported but not enough to meet moderate or high levels) [22]. The moderate- and high-intensity categories were collapsed and a binary indicator reflecting low (yes/no) levels physical activity created. Unhealthy diets were examined using two measures: (1) high fast-food consumption; and (2) sugar-sweetened beverage (SSB) intake. High fast-food consumption was defined as self-reported eating at fast food places such as Burger King, KFC, Tastee, Juici Patties or Pizza Hut at least twice per week. This definition has been used in previous studies assessing excessive fast food consumption [23]. SSBs were defined as soft drinks (i.e., sodas), fruit drinks (i.e., box drinks) and lemonade. In keeping with previous studies [24, 25], high SSB intake included those who reported drinking lemonade/soda/box drink at least daily.

##### Biological factors

Biological factors included sex (i.e., male or female) and age, which was treated as a continuous variable and included all persons 15–74 years.

##### Health system factors

To gauge access to health care services, a binary indicator was created to reflect participant ownership of private insurance. Timing since the respondent last had his/her blood pressure measured was used as a proxy indicator of a recent health service visit. Respondents who reported that their last blood pressure measurement was less than 6 months ago were coded as having a recent health care visit.

##### Statistical approach

A total of the 2848 respondents completed the survey; 311 (10.9%) were missing information on one or more of the 11 NCD indicators and excluded from analysis. There were no statistically significant differences between those with complete (*n* = 2551) versus missing (*n* = 311) data with respect to sex, age or region of residence. Descriptive statistics were used to describe the prevalence of morbidity (from individual NCDs) and multimorbidity across 10-year age-groupings. Means with 95% confidence intervals (for continuous variables), and proportions (for categorial variables) were computed and compared using the one-way analysis of variance (ANOVA) and the Pearson χ^2^ statistic, respectively, to examine differences across age groups. Analyses were weighted using sampling weights, to account for differences in sampling selection probabilities, in addition to post-stratification weights, to account for differences in the age-sex distribution of the study sample compared to the Jamaican population. Base sampling weights reflected the product of the inverse of the probability of selecting a household and the inverse of the probability of selecting a primary sampling unit, adjusted for non-response. Post-stratification weights were calculated as the number of persons in the Jamaican population between the ages of 15–74 years, represented by each individual in the sample within 5-year age-sex categories. In accordance with recommended research practice, however, regressions were unweighted [26].

#### Latent class analysis (LCA)

Probabilistic LCA models facilitate exploration of population heterogeneity by dividing the population into mutually exclusive and exhaustive latent classes (i.e., NCD multimorbidity patterns), based on responses to a set of measured items [17], (i.e., the presence/absence of 11 NCDs). This involved fitting a series of models to the data, starting with a one-class model and increasing classes in a stepwise fashion (i.e., up to 6 classes) until model fit no longer significantly improved. To determine the baseline model, several indices were compared, including the likelihood-ratio G^2^ statistic, the Akaike Information Criteria (AIC), the Bayesian Information Criteria (BIC) and the adjusted BIC, with lower values suggestive of a more optimal fit [17]. Probability plots for the latent classes were also inspected to consider the substantive interpretability (i.e., the meaningfulness and distinctiveness) of the resultant latent class solutions [17].

#### Multinomial logistic regression models

Once the baseline model was identified, participants were assigned to their best fit class based on their maximum posterior probability of class membership. Each class was treated as a dependent variable and analyses performed using multinomial logistic regression. Multinomial logit models are used for variables which lack a natural ordering and simultaneously estimate the effects of the independent variables, for all but one outcome which is selected to reflect the baseline reference category. In this study, the *Relatively Healthy* class was selected as the base category to which other multimorbidity patterns were compared. Regression coefficients were exponentiated to yield relative risk ratios (RRR) and 95% confidence intervals (95% CI), for a unit change in the predictor variable, reflecting the relative risk or the ratio of the probability of membership in one outcome category over the probability of membership in the baseline *Relatively Healthy* class.

As a first step towards building multivariate models, unadjusted multinomial regression analyses were conducted for each SDH to examine the crude relationship of each indicator with the identified latent classes. This step allowed for description of the social composition of identified patterns as well as identification of significant predictors for inclusion in subsequent multivariate models. Any variable found to be a significant predictor of one or more multimorbidity patterns was included in the final model. The absence of multi-collinearity was also tested, using the variance inflation factor (VIF), to ensure robustness of the regression models. Then, guided by the WHO CSDH framework [3] (Fig. 1), a series of multivariate multinomial logistic regression models were run to control for the potentially confounding effects of other indicators. Authors of the CSDH model note that while intermediary determinants are closely tied to both socio-economic position and health outcomes, the association between socio-economic position and health is often reduced, though not eliminated, upon statistically controlling for these determinants [3]. Accordingly, multivariate regression models were performed as a series of steps, where Model 1 included structural determinants (i.e., socio-economic position) only, Model 2 included intermediary determinants only, and Model 3 included all SDH.

As a final step towards ensuring that the multinomial regression was well-specified, the independence of irrelevant alternatives (IIA) test was performed. Generally speaking, multinomial models assume that outcome categories of the model have the property of IIA, meaning that inclusion or exclusion of categories does not affect the relative risks associated with the regressors in the remaining categories [27, 28]. Under the IIA assumption, no systematic change in the coefficients is expected upon excluding one of the outcomes (i.e., one of the multimorbidity classes) from the model [27, 28]. To test the IIA assumption, a Hausman test was performed by first estimating the full multinomial model (i.e., Model 3) with all multimorbidity latent classes and then estimating a restricted model, with one of the outcomes excluded.

All statistical analyses were carried out using Stata v.15 software, with a value of *p* < 0.05 regarded as statistically significant.

## Results

### Sample description

Descriptive statistics for the weighted sample, according to age-group, are presented in Table 2.

**Table 2:** Prevalence of Select Non-Communicable Diseases (NCDs) by age-group (JHLS-II data, 2007/2008; N = 2551)

	Age grouping (%)						
	15–24 (***n*** = 464)	25–34 (***n*** = 519)	35–44 (***n*** = 531)	45–54 (***n*** = 475)	55–64 (***n*** = 296)	65–74 (***n*** = 266)	All ages (N = 2551)
*NCD Prevalence*							
Hypertension***	6.43	11.38	22.97	47.31	59.66	67.03	25.33
Obesity***	12.39	24.12	32.55	34.74	30.11	27.66	25.23
Hypercholesterolemia***	3.85	7.25	12.18	19.50	22.18	24.21	11.53
Diabetes mellitus***	1.12	2.32	6.89	14.28	18.02	30.37	7.86
Asthma*	9.79	6.23	6.66	6.38	3.48	3.64	6.85
Arthritis***	0.13	1.13	3.16	5.75	18.04	29.01	5.24
Cardiovascular disease***	1.73	1.66	3.53	6.67	11.03	17.42	4.62
Mental health disorders	2.95	3.01	3.31	2.84	3.52	1.25	2.96
COPD	2.52	2.94	2.08	4.79	2.66	2.04	2.80
Stroke***	0.18	0.41	0.47	2.08	3.27	6.16	1.21
Glaucoma***	0.00	0.11	0.99	0.75	3.08	7.33	1.10
Multimorbidity (2+ NCDs)***	7.44	10.50	24.16	42.13	51.12	64.01	24.05
Multimorbidity (3+ NCDs)***	0.89	4.13	8.06	19.67	25.29	35.56	10.16
Mean number of NCDs reported (95% CI)***	0.41 (0.34, 0.48)	0.61 (0.52, 0.70)	0.95 (0.85, 1.04)	1.45 (1.30, 1.60)	1.75 (1.58, 1.92)	2.16 (1.98, 2.34)	0.95 (0.90, 0.99)

COPD = chronic obstructive pulmonary disease

Note: Values are weighted proportions (%) or mean (95% CI)

*p*-value for difference across age-groups based on chi-squared (χ^2^) test or one-way analysis of variance (ANOVA), as appropriate (*p < 0.05; **p < 0.01; ***p < 0.001)

There were significant differences across 10-year age-groupings for all NCDs, except mental health disorders and COPD. NCD prevalence generally increased with advancing age, with the exception of asthma where slightly lower prevalence was observed across older age-groups. Nearly, one-quarter (24.05%) of the sample population was multimorbid (i.e., had two or more diseases). Similar to the prevalence of individual NCDs, the burden of multimorbidity significantly increased with advancing age (15–24 years = 7.44% vs. 25–34 years = 10.50% vs. 35–44 years = 24.16% vs. 45–54 years = 42.13% vs. 55–64 years = 51.12% vs. 65–74 years = 64.01%; *p* < 0.001). The mean number of NCDs reported also increased with successive age-groups (*p* < 0.001). Using a more discriminating definition of multimorbidity (i.e., three or more diseases), significant differences across age-groups were also observed with a prevalence of 0.89% among those 15–24 years of age compared to 35.56% prevalence in the 65–74-year age group (*p* < 0.001).

### LCA results

LCA results have been previously described [16]. To summarize, indices and probability plots collectively supported the four-class model as the optimal baseline model, based on identification (i.e., the model converged on the same solution the majority of the time), entropy (i.e., distinctiveness of the latent classes) and meaningful interpretation. The final model comprised 4 classes: a *Relatively Healthy* class (52.70%) with little to no morbidity; a *Metabolic* class (30.88%) characterized by high likelihood of self-reporting hypertension and obesity; a *Vascular-Inflammatory* class (12.21%) characterized by individuals with a high probability of self-reporting hypertension, obesity, diabetes, hypercholesterolemia, arthritis and cardiovascular disease; and a *Respiratory* class (4.20%) characterized by increased likelihood of self-reporting asthma and COPD.

### Social composition of multimorbidity patterns

There were significant differences in the social patterning of multimorbidity according to structural determinants and socio-economic position (i.e., education level, employment status, wealth quintile) and intermediary determinants of health (i.e., proximity to destinations, home environment quality, current alcohol use, past or current smoking status, low physical activity levels, excessive fast-food consumption, SSB intake, insurance coverage, recent health service use). Table 3 shows the social characteristics of each class, as well as the bivariate association of each determinant with the three multimorbidity patterns.

**Table 3:** Respondent characteristics and bivariate association of social determinants of health with multimorbidity patternsǂ (JHLS-II data, 2007/2008; N = 2551)^ǂ^

	Relatively Healthy (***n*** = 1523)		Metabolic (***n*** = 717)		Vascular-Inflammatory (=254)		Respiratory (***n*** = 57)	
	%	RR	%	RR	%	RR	%	RR
*Structural determinants*								
Socio-economic position								
Secondary level or higher	77.73	1.00	50.40	0.28*** (0.23, 0.34)	39.01	0.18*** (0.13, 0.24)	77.72	0.85 (0.48, 1.52)
Employed full-time	44.33	1.00	48.48	0.96 (0.80, 1.15)	31.77	0.60*** (0.46, 0.80)	60.52	1.72* (1.01, 2.94)
Top 60% wealth quintile	66.61	1.00	62.89	0.81* (0.68, 0.97)	61.59	0.80 (0.61, 1.04)	74.74	1.32 (0.75, 2.33)
*Intermediary determinants*								
Material Circumstances								
Housing conditions (mean, 95% CI)	10.60 (10.20, 11.00)	1.00	11.06 (10.60, 11.50)	1.05** (1.02, 1.08)	11.42 (10.93, 11.91)	1.09*** (1.04, 1.14)	10.86 (9.73, 11.99)	1.04 (0.96, 1.14)
Place to obtain fruit & vegetables (in walking distance)	55.19	1.00	54.33	0.89 (0.74, 1.07)	46.43	0.63** (0.48, 0.84)	52.41	0.79 (0.46, 1.36)
Recreational areas in walking distance	49.15	1.00	49.24	0.91 (0.75, 1.10)	41.89	0.69* (0.52, 0.92)	55.85	1.06 (0.62, 1.82)
Greater perceived safety	64.64	1.00	72.96	1.17 (0.97, 1.42)	69.65	1.31 (0.97, 1.77)	72.35	1.39 (0.76, 2.52)
Neighbourhood infrastructure score (mean, 95% CI)	5.22 (5.00, 5.44)	1.00	5.16 (4.93, 5.40)	0.98 (0.93, 1.04)	5.16 (4.89, 5.44)	0.96 (0.88, 1.04)	5.26 (4.90, 5.63)	0.99 (0.84, 1.17)
Behavioural risk factors								
Currently use alcohol	69.41	1.00	57.53	0.57*** (0.48, 0.68)	44.14	0.42*** (0.32, 0.55)	69.94	1.04 (0.60, 1.80)
Past or present smoker	30.62	1.00	34.71	1.22* (1.01, 1.48)	32.43	1.11 (0.82, 1.48)	24.84	0.83 (0.44, 1.56)
Low levels of physical activity	52.15	1.00	52.46	1.09 (0.91, 1.30)	64.29	1.69*** (1.28, 2.24)	75.38	2.04* (1.15, 3.63)
Excessive fast-food consumption	13.94	1.00	6.40	0.33*** (0.22, 0.48)	4.33	0.21*** (0.10, 0.44)	5.60	0.40 (0.12, 1.29)
Consumes SSB at least once daily	50.27	1.00	49.31	0.93 (0.78, 1.12)	39.45	0.70** (0.53, 0.91)	57.35	0.90 (0.53, 1.54)
Biological risk factors								
Age (mean, 95% CI)	31.98 (31.57, 32.40)	1.00	46.07 (45.03, 47.11)	1.08*** (1.07, 1.08)	56.51 (54.66, 58.36)	1.13*** (1.11, 1.14)	37.90 (33.40, 42.39)	1.04***(1.02, 1.06)
Female sex	49.53	1.00	48.71	1.08 (0.90, 1.31)	74.86	2.48*** (1.76, 3.50)	69.92	3.09** (1.45, 6.58)
Health System Factors								
Insurance coverage	18.82	1.00	16.13	0.76* (0.59, 0.98)	27.05	1.61** (1.18, 2.20)**	25.76	1.16 (0.59, 2.28)
Recent health service use	39.08	1.00	54.95	2.01*** (1.67, 2.41)	82.57	6.75*** (4.73, 9.64)	79.56	4.05*** (2.16, 7.60)

CI = confidence interval; RR = risk ratio

ǂFor the above patterns, the *Relatively Healthy* class is characterized by little to no probability of any NCD morbidity; the *Metabolic* class by high probability of hypertension and obesity; the *Vascular-Inflammatory* class by high probability of hypertension, obesity, diabetes, hypercholesterolemia, arthritis and cardiovascular disease; and the *Respiratory* class by high probability of asthma and COPD

^**ǂ**^ < 10% missing data

(*p < 0.05; **p < 0.01; ***p < 0.001)

With regard to structural determinants and socio-economic position, and compared to those in the *Relatively Healthy* class, individuals in the *Metabolic* class were less educated and less wealthy while individuals in the *Vascular-Inflammatory* class were less educated and less likely to be employed full-time. Individuals in the *Respiratory* class were more likely to be employed full-time than their *Relatively Healthy* counterparts. Regarding intermediary determinants, individuals in the *Metabolic* class were more likely to have better housing conditions, less likely to currently use alcohol, more likely to be a past or present smoker, and less likely to consume fast food excessively, than those in the *Relatively Healthy* class. Individuals in the *Vascular-Inflammatory* class were also more likely to have better housing conditions than their *Relatively Healthy* counterparts, but less likely to live in close proximity (i.e., walking distance) to recreation areas or a place where fruits and vegetables could be obtained. These individuals were also less likely to use alcohol, more likely to report low levels of physical activity, and less likely to partake in either excessive fast-food consumption or daily consumption of SSBs. Compared to those in the *Relatively Healthy* class, individuals in the *Respiratory* class were more likely to report lower levels of physical activity.

Individuals across all multimorbidity classes were generally older than their *Relatively Healthy* counterparts, while those in the *Vascular-Inflammatory* and *Respiratory* classes were more likely to be female. Finally, with regard to health system factors, the *Metabolic* class was less likely, and the *Vascular-Inflammatory* class more likely, to report private insurance coverage, compared to the *Relatively Healthy* class. Recent health service use was consistently reported across all multimorbidity patterns.

### Multinomial logistic regression analyses

The VIF for each variable was below the cut-off value of 10 [29], suggesting that multicollinearity was not a problem in subsequent multivariate models. Results of the multivariate, multinomial regression models are presented in Table 4.

**Table 4:** Multivariate multinomial logistic regression results of multimorbidity patternsǂ and social determinants of health (JHLS-II data, 2007/2008; N = 2551)^ǂ^

	Model 1 – Structural determinants only			Model 2 – Intermediary determinants only			Model 3 - All Social Determinants of Health		
	Metabolic	Vascular-Inflammatory	Respiratory	Metabolic	Vascular-Inflammatory	Respiratory	Metabolic	Vascular-Inflammatory	Respiratory
*Structural determinants*									
Socio-economic position									
Secondary level or higher	0.27*** (0.22,0.33)	0.17*** (0.12,0.22)	0.77 (0.42,1.41)				0.73* (0.56,0.96)	0.87 (0.57,1.35)	1.03 (0.50,2.16)
Employed full-time	0.97 (0.80,1.17)	0.61*** (0.46,0.82)	1.69 (0.99,2.89)				0.90 (0.72,1.13)	0.76 (0.52,1.09)	1.68 (0.92,3.07)
Top 60% wealth quintile	1.16 (0.95,1.42)	1.38* (1.03,1.85)	1.34 (0.74,2.42)				1.18 (0.91,1.53)	1.33 (0.89,1.98)	1.56 (0.77,3.19)
*Intermediary determinants*									
Material Circumstances									
Place to obtain fruit & vegetables (in walking distance)				1.06 (0.83,1.36)	0.86 (0.58,1.27)	0.96 (0.49,1.86)	1.04 (0.81,1.34)	0.84 (0.56,1.25)	0.93 (0.48,1.81)
Recreational areas in walking distance				0.90 (0.70,1.16)	0.72 (0.48,1.07)	0.85 (0.43,1.66)	0.94 (0.73,1.21)	0.71 (0.47,1.07)	0.79 (0.40,1.56)
Home quality (mean, 95% CI)				1.03 (0.99,1.07)	1.03 (0.97,1.09)	1.01 (0.91,1.11)	1.03 (0.99,1.07)	1.01 (0.95,1.08)	0.98 (0.89,1.09)
Behavioural risk factors									
Currently use alcohol				0.81 (0.63,1.02)	0.95 (0.66,1.38)	1.51 (0.81,2.81)	0.79 (0.62,1.01)	0.91 (0.63,1.33)	1.42 (0.76,2.66)
Past or present smoker				1.09 (0.83,1.41)	1.27 (0.84,1.92)	0.54 (0.24,1.22)	1.10 (0.84,1.43)	1.27 (0.84,1.92)	0.58 (0.25,1.33)
Low levels of physical activity				1.05 (0.84,1.31)	1.24 (0.87,1.77)	1.73 (0.92,3.26)	1.04 (0.83,1.30)	1.21 (0.84,1.73)	1.83 (0.97,3.45)
Excessive fast-food consumption				0.85 (0.54,1.36)	0.98 (0.39,2.43)	0.73 (0.21,2.51)	0.85 (0.54,1.35)	0.94 (0.38,2.36)	0.71 (0.21,2.45)
Consumes SSB at least once daily				1.07 (0.86,1.34)	0.91 (0.65,1.29)	1.03 (0.58,1.85)	1.07 (0.86,1.33)	0.89 (0.63,1.26)	1.05 (0.58,1.89)
Biological risk factors									
Age (mean, 95% CI)				1.07*** (1.06,1.08)	1.12*** (1.11,1.14)	1.04*** (1.02,1.07)	1.06*** (1.05,1.08)	1.12*** (1.10,1.14)	1.05*** (1.02,1.07)
Female sex				1.04 (0.79,1.36)	2.65*** (1.64,4.28)	1.77 (0.76,4.13)	1.02 (0.78,1.35)	2.54*** (1.56,4.11)	1.95 (0.83,4.60)
Health System Factors									
Insurance coverage				0.60** (0.44,0.83)	1.33 (0.87,2.03)	0.90 (0.42,1.92)	0.63** (0.45,0.87)	1.32 (0.85,2.06)	0.76 (0.35,1.66)
Recent health service use				1.71*** (1.36,2.16)	3.64*** (2.38,5.56)	3.77*** (1.82,7.81)	1.68*** (1.33,2.12)	3.60*** (2.35,5.51)	3.57*** (1.72,7.41)

ǂFor the above patterns, the *Relatively Healthy* class is characterized by little to no probability of any NCD morbidity; the *Metabolic* class by high probability of hypertension and obesity; the *Vascular-Inflammatory* class by high probability of hypertension, obesity, diabetes, hypercholesterolemia, arthritis and cardiovascular disease; and the *Respiratory* class by high probability of asthma and COPD

^**ǂ**^ < 10% missing data; reference group is the Relatively Healthy class

(*p < 0.05; **p < 0.01; ***p < 0.001)

In the model with structural determinants only (Model 1), higher educational attainment was associated with lower likelihood of membership in either the *Metabolic* (RRR = 0.27; 95% CI: 0.22, 0.33) or *Vascular-Inflammatory* (RRR = 0.17; 95% CI: 0.12, 0.22) class. Further, a significant inverse relationship was observed between fulltime employment (RRR = 0.61, 95% CI: 0.46, 0.82) and membership in the *Vascular-Inflammatory* class, while a significant positive relationship was observed between membership in this class and being among the top 60% wealth quintile (RRR = 1.38; 95% CI: 1.03, 1.85).

In the model with only intermediary determinants (Model 2), significant associations were observed with biological risk factors and health system factors, only. Advancing age was a significant predictor of all three multimorbidity patterns (RRR_Metabolic_ = 1.07, 95% CI: 1.06, 1.08; RRR_Vascular-Inflammatory_ = 1.12, 95% CI: 1.11, 1.14; RRR_Respiratory_ = 1.04, 95% CI: 1.02, 1.07). Being female increased the likelihood of membership in the *Vascular-Inflammatory* (RRR = 2.65, 95% CI: 1.64, 4.28) class. Private insurance coverage (RRR = 0.60, 95% CI: 0.44, 0.83) was associated with lower likelihood of belonging to the *Metabolic* class. All multimorbidity classes were significantly associated with recent health service use (RRR_Metabolic_ = 1.71, 95% CI: 1.36, 2.16; RRR_Vascular-Inflammatory_ = 3.64, 95% CI: 2.38, 5.56; RRR_Respiratory_ = 3.77, 95% CI: 1.82, 7.81).

After adjusting for all SDH (Model 3), advancing age (*p* < 0.001) and recent service use (*p* < 0.001) remained significant independent predictors of all multimorbidity classes. Female sex remained a significant independent predictor of membership in the *Vascular-Inflammatory* class (RRR = 2.54; 95% CI: 1.56, 4.11) while higher educational attainment (RRR = 0.73; 95% CI: 0.56, 0.96) and private insurance coverage (RRR = 0.63; 95% CI: 0.45, 0.87) maintained a significant inverse association with membership in the *Metabolic* class. Neither educational attainment, employment status nor wealth remained significant predictors of membership in the *Vascular-Inflammatory* class in Model 3.

Results of the Hausman test provided no evidence that the IIA assumption had been violated, indicating that the null hypothesis of no systematic difference in coefficients could not be rejected [Hausman statistic χ^2^ = 1.53, df = 32, *p* = 0.999]. In other words, there was no evidence against the correct specification of the multinomial model for multimorbidity patterns in the sample.

## Discussion

This is the first study to examine the social patterning of multimorbidity in Jamaica. Building on identified latent classes [16], this study shows that beyond differences in the type and number of diseases comprising multimorbidity patterns, there are also important social differences associated with class membership. Specifically, the study found that advancing age and recent health service use were significantly associated with all multimorbidity patterns. Being female, lower educational attainment and lack of insurance were also independently associated with prevalent multimorbidity – although the significance varied across patterns.

Most international studies on the social patterning of multimorbidity have focused on structural determinants reflecting socio-economic position, and biological factors. These studies often used simple counts of diseases, with fewer investigations exploring relationships across patterns of co-occurring diseases or with intermediary determinants of health. Nonetheless, evidence from these studies suggests a social gradient to multimorbidity, with higher burden among the socio-economically disadvantaged (i.e., less educated, unemployed, lower wealth quintiles) [6, 30]. In this study, lower educational attainment in both *Metabolic* and *Vascular-Inflammatory* classes (compared to the *Relatively Healthy* class) is consistent with the literature on multimorbidity [9, 30]. However, this difference only remained statistically significant among individuals exhibiting the *Metabolic* multimorbidity pattern, after controlling for all SDH.

Among biological factors, age has been well-established as a strong risk factor for chronic disease multimorbidity [6–8, 18]. Similarly, in this study, advancing age was a significant independent predictor of all three multimorbidity patterns. Notably, while the mean number of NCDs was less than 1 in the population under 45 years, the burden of multimorbidity (defined as 2 or more diseases) increased in this population with increasing 10-year bands (i.e., 15–24 years = 7.44%; 25–34 years = 10.50%; 35–44 years = 24.16%). Further, while the mean age of individuals in the *Metabolic* and *Vascular-Inflammatory* classes was 46.07 years (95% CI: 45.03, 47.11) and 56.51 years (95% CI: 54.66, 58.36), respectively, the age-profile of the *Respiratory* class (mean age: 37.90 years; 95% CI: 33.40, 42.39) suggested that this pattern comprised relatively young adults. This finding reflects the fact that multimorbidity is not solely a problem of old age and reinforces the need for prevention efforts across the life course to curb the accumulation of NCDs and promote healthy ageing. Evidence from the 2011 Jamaican population census confirmed a shift in the age structure, with a decline in the under-15 year old population, an increase in the population over 60 years of age and the largest increase among the old-old (i.e., those 80 years and older) [31]. Investigation of the burden and socio-economic patterning of multimorbidity among elderly populations in Jamaica would represent a useful next step, to further inform healthcare and social services planning and support improved health and quality of life in old age. Indeed, LCA of multimorbidity profiles among German centenarians identified a “low morbidity” class (comprising 36% of the sample) with lower odds of hospitalization and long-term care facility use, highlighting the potential for minimal disease burden and good quality of life even at advanced ages [32].

Consistent with evidence of differences in multimorbidity patterns across sex, this study found that being female was associated with an increased risk of membership in the *Vascular-Inflammatory* class. Other studies in Jamaica [33, 34], and the wider Caribbean region [35], have identified a female preponderance in morbidity from individual NCDs and it is not surprising then that women bear a greater burden of multimorbidity as well. Although this is likely due to a range of factors (such as better health-seeking behaviours facilitating disease diagnosis), causes of the gender disparity remain unclear and require further investigation. Available data (i.e., between 2007 and 2018) from the World Bank database [36], show that life expectancy at birth for all age-groups in Jamaica has remained relatively stable at around 74 years and is slightly higher in women (i.e., 76 years) compared to men (i.e., 73 years); while this longevity may allow for development of (multiple) NCDs, the greater disease burden, nonetheless, suggests that more in-depth exploration of lifestyle behaviours, social networks, and coping mechanisms among women may shed light on useful strategies to support management of multiple conditions.

With regard to intermediary determinants, material circumstances (i.e., features of neighbourhood environments) did not appear to be significant predictors of multimorbidity. This is somewhat in keeping with an earlier study in Jamaica which found no significant association between some environmental features – namely, perceptions of safety and availability of recreational spaces within walking distance – and individual NCDs (i.e., overweight/obesity and diabetes) [15]. The need for further exploration into whether specific environmental attributes positively influence NCD prevention and management has been noted, in light of commonly observed inconsistency in associations between proximity to recreational areas and more optimal health behaviours [37].

The relationship between insurance coverage and multimorbidity patterns is particularly interesting, with insurance coverage reducing the likelihood of membership in the *Metabolic* class relative to the *Relatively Healthy* class. Ability to purchase private health insurance may reflect increased financial capacity of these individuals and is in keeping with evidence from the literature that the accumulation of multiple morbidities is more common in the socio-economically deprived [6, 9]. On the other hand, private insurance coverage may reflect better access to care and lower likelihood of forgoing health care contact due to financial concerns, thereby facilitating the early recognition, prompt diagnosis and effective management of disease. Studies indicate that insurance coverage is indeed a significant predictor of health care utilization among older men in Jamaica [38]. Improved financial capacity and health care access through insurance coverage likely extends beyond disease detection to medication procurement and follow-up visits to physicians, suggesting the potential role of this enabling factor in multimorbidity control as well.

Increased health care utilization in association with multimorbidity has been consistently documented across studies, with a greater number of medical visits and more frequent hospitalizations among Spanish adults 50 years and older [39], higher odds of hospitalization and emergency department visits among American Medicare beneficiaries [40], and a preference for secondary outpatient care versus primary care among residents in southern China [9]. Consistent with available literature, results from this study indicated significant positive associations, across all multimorbidity patterns, with recent service use, although it is not clear whether this recent health service contact allowed for the diagnosis of conditions or was prompted in response to the burden of managing multiple conditions. Regardless, this finding alludes to the economic burden of multimorbidity faced by individuals and healthcare systems. A recent population-based Canadian study concluded that the relatively small proportion of the population with multimorbidity was responsible for a disproportionately large proportion of total healthcare costs, noting that the observed non-linearity in costs was likely attributed to complexity regarding the degree of multimorbidity and types of disease combinations [41]. Additional research using prospective follow-up to examine how multimorbidity patterns are associated with preventive versus curative health service use, including primary care services, emergency department visits and hospital admissions, could better inform on health-seeking behaviours and risk of adverse events, as well as costs borne to individuals in managing prevalent diseases and preventing the accumulation of new ones. Importantly, health service use in Caribbean settings is often not limited to bio-medical therapies [42], with previous studies indicating that among Jamaican adults, use of herbal remedies (concomitantly with pharmaceutical treatments) is common, particularly for respiratory system ailments and among the uninsured [43]. Appreciation of the role of alternative medicines in preventive care and/or the management of multimorbidity may have important health system implications regarding medication compliance, adherence to physician recommendations and risk of adverse drug events, to inform physician-patient interactions and public policy.

Evidence from the World Bank database suggests improvements in Jamaica’s social and economic development since 2007/2008 [36]. Yet, emergence of COVID-19 in 2020 may have given rise to unintended challenges impacting health care access and management of multiple chronic diseases. Individual chronic conditions (e.g., obesity, diabetes, hypertension) have been associated with greater risk of more dramatic effects (i.e., hospitalization, death) following COVID-19 infection [44]. Importantly, while the impacts of COVID-19 on multimorbidity burden remain unclear, public health experts have noted that some consequences of COVID-19 (e.g., national border closings, food supply disruptions, stay-at-home orders) may be leveraged into opportunities for Caribbean nations to improve future dietary and physical activity behaviours [44]. Investment in education to promote healthy snacking behaviour and nutrition, built environment reinvigoration to encourage safe outdoor physical activity, and production and consumption of local and regionally produced healthy foods have been touted as strategies to improve disease outcomes and minimize negative impacts, particularly among poor, marginalised groups [44, 45]. In this vein, government-led interventions in Jamaica have focused on improving access to and knowledge of healthy lifestyle behaviours, via community-based farmers markets, distribution of fixed priced ‘vegetable baskets’ to support the agricultural sector, and television and social media-based exercise programmes that aim to minimize potential COVID-19 related amplification of health inequities [44].

### Strengths & Limitations

Although use of the nationally, representative JHLS-II survey data is among the strengths of this analysis, there are several study limitations to be acknowledged. First, the cross-sectional study design limits causal interpretation and reverse causality (i.e., whether disease affected behaviour or behaviour affected disease) remains a plausible explanation for associations identified. For example, the direction of association between health service use and multimorbidity may be unclear, as it is plausible that more recent health service use is a marker of better health-seeking behaviour which would facilitate diagnosis of additional diseases and greater awareness of multiple conditions. Self-reported presence/absence of disease may also be subject to error and may have introduced bias [46]. Interestingly, although mental health disorders, for example, are often underreported due to undiagnosed illness and/or reluctance to disclose, a recent systematic review and meta-analysis on multimorbidity did not identify differences in findings across studies that included or excluded mental health disorders; however, overestimation of associations between specific SDH (i.e., education) and multimorbidity burden has been reported in studies based on subjective (i.e., self-reported) versus objective measurement methods [30]. SDH examined in this study were subject to data availability and there are limitations regarding both the definition and measurement of certain indicators as well as residual confounding due to variables not included in analyses. Features of the neighbourhood environment were not based on formal or validated scales. Convenience measures used could have influenced the lack of significant associations observed while proximity indicators are limited as (walking) distance may not be the sole determinant of use of services and amenities. The interviewer’s subjective interpretation of the respondent’s home and community environments must also be considered; however, among the strengths of the JHLS-II survey, rigorous training and certification of interviewers was ensured prior to field work, and duplicate measures were made by supervisors to ensure data quality, with evidence that good inter- and intra-observer reliabilities were maintained throughout the survey [13]. Yet, it is noted that perceptions of quality and proximity may differ between the respondent and interviewer, and an interviewer-based evaluation may not accurately reflect individual-level perceptions, motivations and circumstances. Cronbach’s alpha coefficients support the internal consistency of the summative scales used while their use in a previous local study [15], allows for comparability and builds on previous research. High non-response rates for some questionnaire items (e.g., household income) precluded their inclusion for analytical examination. Although a proxy indicator of wealth was created based on a weighted composite of measures (i.e., non-productive assets and living conditions), limitations of such an index are noted, including failure to capture asset quality/value, short-term interruptions and household shocks [47]. Finally, potentially informative SDH, such as psychosocial factors (e.g., social interaction, social support) and material circumstances (e.g., number of children living with respondent) were omitted from the analysis since questionnaire items did not allow for operationalization of these concepts. The role of family structure in multimorbidity prevalence and control remains relatively understudied, and evidence on the role of family obligations and social networks is mixed [48–50]. A recent study among older Jamaican men identified regular attendance at club (e.g., society, religious) meetings and financial support from a son or daughter as significant predictors of prostate checks and routine doctors’ visits, respectively [38]. Social support systems may thus play an important role in preventive service uptake, and further exploration is needed to determine whether these networks can be leveraged to support the effective management of diseases and timely detection of complications that would halt the accumulation of multiple conditions. As with other latent variable models, misclassification error is reasonable with LCA, particularly with assignment of individuals to a best fit class using posterior probabilities; a method which does not account for classification uncertainty [17]. This limitation should be borne in mind in interpretation of the results. Yet, with approximately 190 patterns observed in the sample, the LCA model approach reduced data complexity, facilitating parsimony and interpretation via identification of population subgroups with similar disease probability profiles. Lastly, due to unequal class sizes and the smaller sub-sample of male (compared to female) respondents, the study was not powered to analyse the social patterning of multimorbidity separately, according to sex.

## Conclusions

This study highlights differences in the social composition of multimorbidity classes in Jamaica, allowing for a more nuanced understanding of the socio-demographic profiles of multimorbidity patterns than would have been possible with simple counts of diseases. Overall, individuals with multimorbidity were older, female, less educated, and uninsured, with greater healthcare use. Future work should explore the social patterning of multimorbidity via longitudinal designs to better identify social factors implicated in the accumulation of multiple conditions and understand how individuals modify their behaviours to manage their multimorbidity. Furthermore, exploration of the relationship of various multimorbidity patterns with health care spending, emergency department visits and hospital admissions can provide targeted information for program planning, resource allocation, improved disease management and reduced complications. Given the potential of multimorbidity to erode financial security and compromise self-care capacity through the burden and complexity of managing multiple diseases, the impact of multimorbidity patterns on quality-of-life outcomes should also be investigated.

## Data Availability

The datasets generated and/or analysed during the current study are not publicly available as the authors are still using the data for other analyses. However, data will be made available from the corresponding author upon reasonable request.

## Abbreviations

AIC: Akaike Information Criteria
BIC: Bayesian Information Criteria
CD: Compact disk
COPD: Chronic obstructive pulmonary disease
CSDH: Commission on Social Determinants of Health
IIA: Independence of irrelevant alternatives
IPAQ: International Physical Activity Questionnaire
JHLS-II: Jamaica Health and Lifestyle Survey 2007/2008
LCA: Latent class analysis
MET: Metabolic equivalent
NCD: Non-communicable disease
OR: Odds ratio
PCA: Principal components analysis
RRR: Relative risk ratio
SDH: Social determinants of health
SSB: Sugar-sweetened beverage
VIF: Variance inflation factor
WHO: World Health Organization

## References

[1] World Health Organization Regional Office for Europe. Gaining Health: The European Strategy for the Prevention and Control of Noncommunicable Diseases. 2006. Copenhagen Ø, Denmark http://www.euro.who.int/document/E89306.pdf. Accessed 27 Jan 2021.

[2] Alwan A (2011). Global status report on noncommunicable diseases 2010.

[3] Solar O, Irwin A. A conceptual framework for action on the social determinants of health. 2010 http://apps.who.int/iris/bitstream/10665/44489/1/9789241500852_eng.pdf?ua=1&ua=1. Accessed 16 Oct 2015.

[4] World Health Organization. Multimorbidity: Technical Series on Safer Primary Care. 2016. Geneva, Switzerland: World Health Organization https://eprints.gla.ac.uk/133210/1/133210.pdf. Accessed 9 Mar 2015.

[5] Johnston MC, Crilly M, Black C, Prescott GJ, Mercer SW (2019). Defining and measuring multimorbidity: a systematic review of systematic reviews. Eur J Pub Health.

[6] Violan C, Foguet-Boreu Q, Flores-Mateo G, Salisbury C, Blom J, Freitag M, Glynn L, Muth C, Valderas JM (2014). Prevalence, determinants and patterns of multimorbidity in primary care: a systematic review of observational studies. PLoS One.

[7] Prados-Torres A, Calderón-Larrañaga A, Hancco-Saavedra J, Poblador-Plou B, Van Den Akker M (2014). Multimorbidity patterns: a systematic review. J Clin Epidemiol.

[8] Nguyen H, Manolova G, Daskalopoulou C, Vitoratou S, Prince M, Prina AM (2019). Prevalence of multimorbidity in community settings: A systematic review and meta-analysis of observational studies. J Comorb.

[9] Wang HH, Wang JJ, Wong SY, Wong MC, Li FJ, Wang PX (2014). Epidemiology of multimorbidity in China and implications for the healthcare system: cross-sectional survey among 162,464 community household residents in southern China. BMC Med.

[10] Alaba O, Chola L (2013). The social determinants of multimorbidity in South Africa. Int J Equity Health.

[11] Ferguson TS, Younger NOM, Tulloch-Reid MK, Lawrence Wright MB, Ward EM, Ashley DE (2008). Prevalence of prehypertension and its relationship to risk factors for cardiovascular disease in Jamaica: analysis from a cross-sectional survey. BMC Cardiovasc Disord.

[12] Ferguson TS, Tulloch-Reid MK, Younger NO, McFarlane SR, Francis DK, Wilks RJ (2011). Prehypertension in Jamaica: a review of data from recent studies. West Indian Med J.

[13] Wilks R, Younger N, Tulloch-Reid M, McFarlane S, Francis D. Jamaica Health and Lifestyle Survey II. 2018. Kingston: Tropical Medicine Research Institute, University of the West Indies https://www.moh.gov.jm/wp-content/uploads/2015/05/Jamaica-Health-and-Lifestyle-Survey-2007-8.pdf. Accessed 16 Oct 2015.

[14] Ferguson TS, Francis DK, Tulloch-Reid MK, Younger NOM, McFarlane SR, Wilks RJ (2011). An update on the burden of cardiovascular disease risk factors in Jamaica: findings from the Jamaica health and lifestyle survey 2007-2008. West Indian Med J.

[15] Cunningham-Myrie CA, Theall KP, Younger NO, Mabile EA, Tulloch-Reid MK, Francis DK, McFarlane SR, Gordon-Strachan GM, Wilks RJ (2015). Associations between neighborhood effects and physical activity, obesity, and diabetes: the Jamaica health and lifestyle survey 2008. J Clin Epidemiol.

[16] Craig LS, Hotchkiss DR, Theall KP, Cunningham-Myrie C, Hernandez JH, Gustat J (2020). Prevalence and patterns of multimorbidity in the Jamaican population: a comparative analysis of latent variable models. PLoS One.

[17] Lanza S, Rhoades B (2013). Latent class analysis: an alternative perspective on subgroup analysis in prevention and treatment. Prev Sci.

[18] Diederichs C, Berger K, Bartels DB (2011). The measurement of multiple chronic diseases - A systematic review on existing multimorbidity indices. J Gerontol A Biol Sci Med Sci.

[19] Kyle TK, Dhurandhar EJ, Allison DB (2016). Regarding obesity as a disease: evolving policies and their implications. Endocrinol Metab Clin N Am.

[20] Tavakol M, Dennick R (2011). Making sense of Cronbach’s alpha. Int J Med Educ.

[21] Kerr J, Rosenberg D, Frank L (2012). The role of the built environment in healthy aging: community design, physical activity, and health among older adults. J Plan Lit.

[22] IPAQ Research Committee. Guidelines for Data Processing and Analysis of the International Physical Activity Questionnaire (IPAQ) - Short and Long Forms. 2005. http://www.ipaq.ki.se/scoring. pdf. Accessed 25 Nov 2018.

[23] Laxer RE, Janssen I (2014). The proportion of excessive fast-food consumption attributable to the neighbourhood food environment among youth living within 1 km of their school. Appl Physiol Nutr Metab.

[24] Malik VS, Schulze MB, Hu FB (2006). Intake of sugar-sweetened beverages and weight gain: a systematic review. Am J Clin Nutr.

[25] Schulze MB, Manson JE, Ludwig DS, Colditz GA, Stampfer MJ, Willett WC, Hu FB (2004). Sugar-sweetened beverages, weight gain, and incidence of type 2 diabetes in young and middle-aged women. JAMA..

[26] Gelman A (2007). Struggles with survey weighting and regression modeling. Stat Sci.

[27] Hausman J, Mcfadden D (1984). Specification tests for the multinomial logit model. Econometrica..

[28] Statacorp (2017). Stata: Release 15. Statistical Software.

[29] Wooldridge JM (2009). Introductory Econometrics: A Modern Approach.

[30] Pathirana TI, Jackson CA (2018). Socioeconomic status and multimorbidity: a systematic review and meta-analysis. Aust N Z J Public Health.

[31] Eldemire-Shearer D, Mitchell-Fearon K, Laws H, Waldron N, James K, Holder-Nevins DL (2014). Ageing of Jamaica’s population – what are the implications for healthcare?. West Indian Med J..

[32] Gellert P, von Berenberg P, Zahn T, Neuwirth J, Kuhlmey A, Dräger D (2019). Multimorbidity profiles in German centenarians: a latent class analysis of health insurance data. J Aging Health.

[33] Mitchell-Fearon K, Waldron N, James K, Laws H, Holder-Nevins D, Eldemire-Shearer D (2014). Hypertension and diabetes prevalence in older persons in Jamaica, 2012. West Indian Med J..

[34] Ferguson TS, Tulloch-Reid MK, Gordon-Strachan G, Hamilton P, Wilks RJ (2012). National health surveys and health policy: impact of the Jamaica health and lifestyle surveys and the reproductive health surveys. West Indian Med J..

[35] Sobers-Grannum N, Murphy MM, Nielsen A, Guell C, Samuels TA, Bishop L, Unwin N (2015). Female gender is a social determinant of diabetes in the Caribbean: a systematic review and meta-analysis. PLoS One.

[36] The World Bank. World development indicators database. 2019. http://datatopics.worldbank.org/world-development-indicators/. Accessed 19 Apr 2019.

[37] Cunningham-Myrie CA, Royal-Thomas TYN, Bailey AE, Gustat J, Theall KP, Harrison JE (2019). Use of a public park for physical activity in the Caribbean: evidence from a mixed methods study in Jamaica. BMC Public Health.

[38] Willie-Tyndale D, McKoy Davis J, Holder-Nevins D, Mitchell-Fearon K, James K, Waldron NK, Eldemire-Shearer D (2019). Predictors of health service utilization among older men in Jamaica. J Gerontol B Psychol Sci Soc Sci.

[39] Olaya B, Victoria Moneta M, Félix Caballero F, Tyrovolas S, Bayes I, Luis Ayuso-Mateos J (2017). Latent class analysis of multimorbidity patterns and associated outcomes in Spanish older adults: a prospective cohort study. BMC Geriatr.

[40] Whitson HE, Johnson KS, Sloane R, Cigolle CT, Pieper CF, Landerman L, Hastings SN (2016). Identifying patterns of multimorbidity in older Americans: application of latent class analysis HHS public access. J Am Geriatr Soc.

[41] Thavorn K, Maxwell CJ, Gruneir A, Bronskill SE, Bai Y, Koné Pefoyo AJ, Petrosyan Y, Wodchis WP (2017). Effect of socio-demographic factors on the association between multimorbidity and healthcare costs: a population-based, retrospective cohort study. BMJ Open.

[42] Aarons DE (1999). Medicine and its alternatives health care priorities in the Caribbean. Hast Cent Rep.

[43] Picking D, Younger N, Mitchell S, Delgoda R (2011). The prevalence of herbal medicine home use and concomitant use with pharmaceutical medicines in Jamaica. J Ethnopharmacol.

[44] Oni T, Micklesfield LK, Wadende P, Obonyo CO, Woodcock J, Mogo ERI, Odunitan-Wayas FA, Assah F, Tatah L, Foley L, Mapa-Tassou C, Bhagtani D, Weimann A, Mba C, Unwin N, Brugulat-Panés A, Hofman KJ, Smith J, Tulloch-Reid M, Erzse A, Shung-King M, Lambert EV, Wareham NJ (2020). Implications of COVID-19 control measures for diet and physical activity, and lessons for addressing other pandemics facing rapidly urbanising countries. Glob Health Action.

[45] Murphy MM, Guariguatax L, Samuels TA (2020). A COVID-19 opportunity: applying a systems approach to food security and noncommunicable diseases. Rev Panam Salud Publica.

[46] Fortin M, Lapointe L, Hudon C, Vanasse A, Ntetu AL, Maltais D (2004). Multimorbidity and quality of life in primary care: a systematic review. Health Qual Life Outcomes.

[47] Vyas S, Kumaranayake L (2006). Constructing socio-economic status indices: how to use principal components analysis. Health Policy Plan.

[48] Shippee ND, Shah ND, May CR, Mair FS, Montori VM (2012). Cumulative complexity: a functional, patient-centered model of patient complexity can improve research and practice. J Clin Epidemiol.

[49] Agborsangaya CB, Lau D, Lahtinen M, Cooke T, Johnson JA (2012). Multimorbidity prevalence and patterns across socioeconomic determinants: a cross-sectional survey. BMC Public Health.

[50] Taylor AW, Price K, Gill TK, Adams R, Pilkington R, Carrangis N (2010). Multimorbidity - not just an older person’s issue. Results from an Australian biomedical study. BMC Public Health.

